# Detectability in Audio-Visual Surveys of Tropical Rainforest Birds: The Influence of Species, Weather and Habitat Characteristics

**DOI:** 10.1371/journal.pone.0128464

**Published:** 2015-06-25

**Authors:** Alexander S. Anderson, Tiago A. Marques, Luke P. Shoo, Stephen E. Williams

**Affiliations:** 1 Centre for Tropical Biodiversity and Climate Change, College of Marine & Environmental Sciences, James Cook University, Townsville, Queensland, Australia; 2 Research Unit for Wildlife Population Assessment, Centre for Research into Ecological and Environmental Modeling, University of St Andrews, The Observatory, Buchanan Gardens, St Andrews, Scotland; 3 School of Biological Sciences, The University of Queensland, St. Lucia, Queensland, Australia; Università degli Studi di Milano-Bicocca, ITALY

## Abstract

Indices of relative abundance do not control for variation in detectability, which can bias density estimates such that ecological processes are difficult to infer. Distance sampling methods can be used to correct for detectability, but in rainforest, where dense vegetation and diverse assemblages complicate sampling, information is lacking about factors affecting their application. Rare species present an additional challenge, as data may be too sparse to fit detection functions. We present analyses of distance sampling data collected for a diverse tropical rainforest bird assemblage across broad elevational and latitudinal gradients in North Queensland, Australia. Using audio and visual detections, we assessed the influence of various factors on Effective Strip Width (ESW), an intuitively useful parameter, since it can be used to calculate an estimate of density from count data. Body size and species exerted the most important influence on ESW, with larger species detectable over greater distances than smaller species. Secondarily, wet weather and high shrub density decreased ESW for most species. ESW for several species also differed between summer and winter, possibly due to seasonal differences in calling behavior. Distance sampling proved logistically intensive in these environments, but large differences in ESW between species confirmed the need to correct for detection probability to obtain accurate density estimates. Our results suggest an evidence-based approach to controlling for factors influencing detectability, and avenues for further work including modeling detectability as a function of species characteristics such as body size and call characteristics. Such models may be useful in developing a calibration for non-distance sampling data and for estimating detectability of rare species.

## Introduction

Worldwide, many bird species may be at risk of decline or extinction [1]. This problem is particularly acute in the montane tropics, where high levels of diversity and endemism are coupled with increased vulnerability to changes in climate and land use [2,3]. Detecting and understanding changes in population size and distribution is crucial to planning appropriate conservation strategies, and depends on accurate information on patterns of density in space and time [4,5]. As a complete census of animal populations is typically unachievable in natural systems [6], most monitoring programs and ecological studies use fixed area or effort counts that generate an index of relative abundance [7]. While such indices are popular and relatively simple to apply, detection probability may vary widely between individuals, survey locations, or times [8], biasing estimates of density such that underlying ecological processes are difficult to infer [6,9].

A variety of survey approaches have been proposed to model detectability, i.e. to correct the number of animals detected accounting for those present but missed (reviewed in [6]). Line transect distance sampling uses the distribution of the *n* detected distances *x* perpendicular to the transect to model detectability as function of perpendicular distance, referred to as the detection function g(*x*) [10]. The detection function is usually modeled via dedicated software [11] and used to estimate the average detection probability *P*, which allows to scale up the number of detected animals to the total number of animals (see [10] for details). Density in the covered area of size *a*, *D*, is then estimated by *n/aP*. An equivalent expression is *n*/2 L ESW, where ESW is the effective strip (half)-width. If all animals up to distance ESW were detected we would detect on average as many animals as were actually detected up to a truncation distance *w* [11]. This makes the correspondence between the actual distance sampling survey and an equivalent strip transect leading to the same number of detections. Given a set of transects randomly distributed in the environment, unbiased distance sampling estimates require a set of assumptions to hold to a reasonable extent: 1: probability of detection along the midline is certain, 2: accurate measurement of distances, and 3: that the observation process is instantaneous. The later assumption is essentially about animal movement, and provided the observer speed is not significantly lower than the animal speed, no problems should arise. If animal speed is faster than observer speed and especially if undetected or responsive movements occur, problems can be anticipated [10]. Whereas surveys in open environments and of large taxa may satisfy these assumptions [12], in more complex environments and with more cryptic taxa problems might arise [13,14]. High diversity, complex habitats and difficulty in access can also add additional challenges in tropical rainforests [14–16].

There is a substantial literature on optimizing Distance survey methods, from point counts to line-transects (see e.g.: [6,13,17]) Much of the debate surrounds the trade-off between maximizing detection probability, optimizing the ratio of survey to transit time, and minimizing the influence of movements and/or double counting. In fact there is no single optimal approach for all cases; some recommend short counts to reduce bias from animal movements [17], while others have shown point counts may give more biased estimates than line transects [14,18] or that the latter increase detection probability [19]. The ratio of survey to transit time is also increased in line transect designs [17] which may thus be the most efficient and least biased method. However, regardless of the duration and length of the survey, whenever the ultimate goal is accurate estimation of population density, the relationship between a) what is present in the survey area and b) what is actually detected, is of critical importance. Rather than adding further to the debate on which survey distance or duration is optimal in a particular case, here we focus on identifying some factors that influence detectability in tropical forests.

A sustained program of biodiversity monitoring, including extensive avian count data to which this study contributed, has established the Australian Wet Tropics among the better-studied tropical rainforest systems, facilitating a range of ecological studies (see e.g. [20,21]). High levels of bird diversity and endemism have also contributed to the listing of montane rainforests in the region among Australia’s “Important Bird Areas” [22], while recent projections of climate change impacts on distributions [23] and populations [24] have highlighted the importance of understanding and monitoring vulnerable species in the region [25]. However, these studies have relied on estimates of relative abundance only, so that covariates of detectability and resulting biases among species, sites and surveys remain unknown. A substantial literature also exists describing such covariates of detectability in bird surveys, in which four main classes can been identified: (1) the characteristics of the objects being detected, (e.g. size of individuals and characteristics of detection cues, [26,27]); (2) the conditions of the survey (e.g., weather; [28]); (3) the characteristics of the location (e.g. habitat structure; [29]); and, (4) the characteristics of the observer (e.g., experience; [30]). Here we focus on covariates applicable to the first three of these, and evaluate the application of distance sampling in the diverse avian assemblage of the Australian Wet Tropics. We identify some important covariates of detection probability and sources of bias in density estimation, and suggest some improvements to protocols for avian surveys in rainforest based on our findings.

## Materials and Methods

### Study area

The study was conducted in the Australian Wet Tropics World Heritage Area (hereafter “AWT”) between 15°45'32.69"S 145°1'53.87"E and 19°18'0.65"S 146° 9'41.17"E. The study region is described in detail in [20] but briefly, rainforests in the AWT occur on coastal ranges of the Great Divide and adjacent lowlands, giving a broad elevational gradient (200 m to ~1600 m above sea level). The structure and floristics of these forests varies from complex mesophyll vine forest in the coastal lowlands to notophyll vine forest and microphyll fern thicket on high peaks and plateaus, though most surveys were conducted in simple to complex notophyll vine forests [31]. Basal area of forests also declines towards the lowlands [32], creating gradients in the structural complexity of vegetation. AWT climate is characterised by warm average temperatures (lowlands: 23.33°C uplands: 19.16°C) and high rainfall (lowlands: 2510 mm, uplands: 2757 mm), concentrated in the summer (based on modeled climate surfaces from BIOCLIM [33]).

### Data collection

All fieldwork was conducted under Queensland Department of Environment and Resource Management research permits, numbers WISP04061506 and WITK04061406. In this study we consider a line transect survey method employed since 1997 to conduct some 1500 surveys during the course of a program of long-term monitoring of rainforest biodiversity in the study region [20,21,24,25]. Line transects have been shown to both maximise the detection of species [19] and minimise bias in density estimation in forest bird surveys [18]. The sampling design is described in detail in [20] but briefly, we surveyed 1000 m sampling routes (“sites”) established in contiguous montane and lowland rainforest. The AWT protects more than 100,000 hectares of steep, mountainous tropical rainforest, in places entirely lacking vehicle access. As in many such studies in the montane tropics [15], this challenging terrain and vegetation often necessitated that long-term monitoring sites be associated with roads and tracks for practical and frequent access. Climate change impacts are also primary focus of the monitoring program, so that the most efficient sampling of the relevant environmental gradients (temperature, rainfall) is achieved by placing surveys at regular intervals across altitude and latitude [20]. Provided care is taken to control or sample for variation in other factors (e.g. vegetation, slope, aspect) stratifying locations in this way provides both efficiency advantages over random placement [10] and statistical advantages over uniform transect placement [34], both critical considerations in designing optimal and cost-effective monitoring.

In this study, 30-minute, 150-metre audio-visual line-transect surveys were conducted at these sites on suitable days between 0600 and 0930 h, in both summer and winter, by a single experienced observer (A. A.). Survey transects were usually placed at 200 m intervals along the 1km site route, and perpendicular to it. In some cases (~25%, see n values in Table 1) steep terrain or dense vegetation meant surveys were conducted along the access road or track, and potential road-induced biases in density estimates (see e.g. [35]) are discussed below.

**Table 1:** A comparison of models incorporating likely detection covariates.

Model	Model factors	AICc	ESW (and 95% CI)	N value per covariate level
*1*	**Species**	**30995.5**	**35.14 (34.49, 35.81)**	**max = 608, min = 6, mean = 46.23**
*2*	**Detection cue**	**31321.71**	**38.80 (38.04, 39.58)**	**seen = 2126, heard = 8286**
*3*	**Body size**	**32148.70**	**38.85 (38.20, 39.51)**	**small = 2258, med = 5843, large = 2763**
***4***	**Distance-only model**	**33223.65**	**44.13 (42.40, 45.92)**	**n = 10412**
*5*	Survey site	33259.37	41.60 (40.95, 42.26)	max = 1267, min = 28, mean = 254
*6*	Site elevation	33340.83	42.05 (41.41, 42.71)	upland = 2395, lowland = 8017
*7*	Survey temperature	33350.42	42.09 (41.44, 42.74)	cool = 3879, warm = 5292
*8*	Survey route	33372.28	42.14 (41.50, 42.80)	forest = 6379, road = 2207
*9*	Survey wetness	33373.07	42.16 (41.51, 42.81)	dry (1) = 4501, wet (2) = 5911
*10*	Bird diversity	33378.77	42.17 (41.53, 42.83)	high = 6157, low = 4255
*11*	Bird abundance	33381.01	42.18 (41.53, 42.83)	high = 5210, low = 5202
*12*	Habitat complexity	33382.97	42.18 (41.54, 42.84)	high = 3053, low = 7259
*13*	Wind	33383.48	42.19 (41.54, 42.84)	high = 1752, low = 8660
*14*	Noise	33384.69	42.18 (41.54, 42.84)	noisy = 1905, quiet = 3472
*15*	Canopy density	33385.20	42.19 (41.54, 42.84)	high = 8363, low = 2049
*16*	Shrub density	33385.41	42.19 (41.55, 42.85)	high = 8375, low = 2037
*17*	Survey season	33385.43	42.19 (41.54, 42.85)	“Wet” = 3319, “Dry" = 7093
*18*	Survey rain	33395.04	42.22 (41.57, 42.87)	high = 1817, low = 8595
*19*	Cluster size	33395.77	42.22 (41.57, 42.87)	single = 9673, group = 775

Models incorporating habitat, weather, temporal and species covariates of the detection function are shown ranked in order of their AIC_c_ score, along with their associated ESW estimates. The model without covariates and all better-performing models are shown in bold. N values for each factor level are shown, except in the case of factors with many levels, in which case the range and mean values are indicated.

The distance sampling methods we use have previously been described in [36,37] but briefly, we measured perpendicular distance from the transect midline to each bird sighting, to the nearest meter, using an Opti-Logic LH400 Laser Range finder (Opti-logic, Tullahoma, TN http://www.opti-logic.com/lh_series.htm). Distances to sighted individuals located ahead of the observer (and therefore not measurable as perpendicular from the transect midline) were estimated, with frequent calibration checks by range finder, as suggested in [38]. Distances were also estimated to all individuals detected by their calls, to the best of the observer’s ability (see schematic in supplementary material, S1 Fig). Importantly, in audio-visual bird surveys the visual detection function tends to decrease more steeply than the aural [39]. This effect is magnified in rainforest, as visual cues rapidly attenuate in their characteristic low light conditions and dense foliage [15,40]. Consequently, audio detections may account for more than 80% of the total [41], and though distances estimated to acoustic cues are more error prone [42], combining data across cue types may be necessary to achieve sufficient sample sizes, especially for rarer species. However, as visual detections may cluster close to the observer [39], the distance/detectability response may thus differ between these and aural cues, so that the resulting “composite” detection function must be fitted with care. In fact, because calls often provide the cues to later locate individuals by sight, audio cues may be even more important in forest bird surveys than suggested by the simple ratio of “seen” versus “heard” detections [43]. As is standard in such surveys, whenever possible we prioritised identification and distance estimation accuracy by visually locating individuals that were heard, especially those nearby the transect. This leads to some initially acoustic detections being “upgraded” to sightings. While here we do not distinguish “heard, then seen” as category (but for an analysis that does so see [43]), we analyzed the detection functions of “heard” and “seen” cue types separately to check for bias resulting from their attenuation patterns (see results below). This indicated whether or not pooling audio and visual detections together produced a reliable estimate of the composite detection function. Birds in flight through the survey area when first detected were excluded from the analysis, while birds flushed by the surveyor were recorded at their estimated original position only. Lastly, where birds were detected in clusters, we also recorded cluster size. When group membership was difficult to assign, we considered each individual as the unit for analysis, instead of a loosely defined group.

Survey duration is an important consideration in the design of distance sampling surveys [10]. For density estimation using the Distance approach, totals ideally represent a snapshot of individuals using the survey area [17], but in practice it is usually impossible to simultaneously enumerate all individuals present, especially in forested habitats [44]. As a result of this delay, movements of individuals during sampling can bias estimates by 1: accumulation of detected individuals as they move into the survey area, 2: double counting of previously detected individuals that have moved ahead to a new location, and 3: attraction or avoidance movements by individuals prior to detection, resulting in non-uniform patterns of density relative to the transect midline. In practice, it may also be difficult to address one of these sources of error without increasing another [44], or compromising other aims of the survey. Here we use a slightly slower rate of movement than in many temperate studies, but one not unusual in tropical forest bird surveys, where difficult terrain and high species diversity demand more time from observers in order to survey sites accurately and thoroughly. Line transects are certainly less prone to bias from the above sources than point counts [44], and we use a “look-ahead” sampling protocol [45] to further reduce bias from movement by 1: allowing birds to be “mapped” ahead of the observer and 2: recorded before they move. We use a ~25 metre visual window and ~60 metre audio window ahead of the observer, and are conservative in recording detections as new individuals. We discuss the consequences of these choices below.

### Distance analysis

Here we are interested in the development of methods that both identify key influences on detection distances in rainforest birds, and maximise the utility of audio-visual surveys for estimating density. For this reason we wish to explore both 1: the importance of covariates overall (a model selection question), and 2: the significance and magnitude of their effect on estimated density for each species. We used Distance software, version 6 [11] to identify important survey and habitat covariates (described below) using an information theoretic approach to model selection, as a precursor to estimating densities and comparing the magnitude of their influence on a per-species basis. While 60 or more observations have been recommended for reliable inferences [10], there are examples of published studies with sample sizes as low as 32 [17], or 20 [46]. As a compromise between including sufficient distances to fit an accurate detection function, or including more species across which to compare covariate effects, here we used a lower threshold of 35 individuals per factor level in each comparison. In the case of transect placement, low numbers of road surveys meant that fewer species met this minimum detection requirement. As we were interested nonetheless to explore the effect of placing surveys along roads, we used a lower threshold of 25 detections per species, but interpret these results with caution. Distance frequency histograms for each species were inspected, and detections closer than 40m binned into 10-m intervals to minimize the effect of “heaping” around commonly estimated distances. To reduce the influence of false precision from larger distances (and hence less accurate distance estimates [42]) detections at distances greater than 40 metres were binned into into 20-m intervals. Histograms were truncated at 100 m to avoid problems in fitting the tail of the detection function [10], which also excluded the least accurate distance estimates. The Akaike Information Criterion adjusted for small sample sizes (AIC_c_) was used to select the most parsimonious model from all possible combinations of Uniform, Half Normal and Hazard Rate models with Cosine, Simple Polynomial and Hermite Polynomial adjustment keys, except in the case of the species covariate models, where we constrained the models to a Half Normal function with Cosine adjustment to achieve consistent convergence.

The cue type covariate compared detections from audio cues against those from visual cues. The body mass covariate compared small (<10 g), medium (10–50 g) and large (>50 g) bodied species, based on mean weights for species from the Handbook of Australian, New Zealand and Antarctic Birds [47]. Species was also analysed as a factor covariate. Cluster size was analysed as a continuous covariate based on observed group sizes. Binary factor covariates for temperature, rain, wind, wetness, and noise were analysed using high or low scores for each survey, relative to the mean value for that site. We avoided surveying in heavy or persistent rain, but nonetheless, “wet” conditions, independent of rain, are a feature of field surveys in rainforest, and here this term refers to moisture in the soil, on leaf surfaces, and dripping from the canopy. The noise covariate was derived from numeric categorical scores for the contribution to background noise levels from wind, rain, canopy drip, nearby streams, and from calling birds and insects. The scoring system we employed is included in the supplementary material (S1 Table). The total number of individuals and total species recorded per transect were calculated to give a covariate for bird species diversity and abundance, which may influence detectability due to observer “swamping” [17,48,49]. Finally, the effect of site habitat structure was examined by including elevation as a factor covariate (upland versus lowland), as well as shrub and canopy layer foliage density scores for each site (high and low density), indexed using a modified Braun-Blanquet method [50]. The need for the inclusion of covariates was assessed by AICc.

Having identified the important covariates of detection, we then analysed data across all species, surveys and sites with the Multiple Covariates Distance Sampling (MCDS) analysis engine [51] in Distance to compare the influence of these covariates on ESW. Here, the number of species compared differs between covariates because sample sizes were determined by the numbers of available detections within each factor level. For each species, we divided the data into subsets for each factor level and fitted separate detection functions to quantify the effect of interactions between species and covariates on ESW. This overcomes a constraint of the MCDS implementation in Distance that limits covariates to influence solely the scale of the detection function, and not its shape [52]. As a non parametric test of the null hypothesis that ESW is the same across factor levels, we conducted Mann-Whitney U-tests (based on the difference in ranking of species’ ESW estimates between factor levels) across all species under each factor level. Since a difference in ESW of a few metres can have proportionally larger impact on density estimation for species with small detection distances, we also expressed differences between factor levels as a proportion of the larger estimate, and compared their means. Lastly, we compared the ESW for each species/factor level combination to look for significant sources of bias, defined as non-overlapping 95% confidence intervals between the estimates of ESW for each level. Non-overlapping 95% confidence intervals are a conservative test of difference [53], which we considered an appropriate gauge of covariate influence in the context of multiple comparisons. Taken together these analyses yield; 1: from AIC_c_ scores, a series of model comparisons (over all species) indicating each covariate’s importance, 2: from Mann-Whitney U-tests, a non-parametric test of this difference, 3: an illustration of the mean magnitude of any bias in density estimation caused by that covariate (as a proportion of ESW) and 4: an indication of the prevalence and magnitude of significant factor effects per species (from 95% confidence intervals). Statistical tests not performed in the Distance software were carried out within the R framework for statistical analysis version 2.13.1 [54].

## Results

A total of 284 distance sampling surveys in the AWT yielded 10,341 bird records across 41 sampling sites. Of these, 8,698 were individual animals and 1,220 clusters of animals. The most often detected species was the Yellow-spotted Honeyeater *(Meliphaga notata*, 608 records*)*, and the rarest was the Russet-tailed Thrush *(Zoothera lunulata*, 6 records), with a mean of 46.2 records per species. A total of 70 species were recorded, 52 of which had 35 or more records which we consider sufficient for analyses at the species level. The number of species with sufficient records per factor level was naturally lower, and varied between factors. Model key function, adjustment terms, and estimates of the ESW, density and mean cluster size across all sites and surveys for each species are provided in S2 Table.

### Characteristics of detected objects

**Fig 1:**
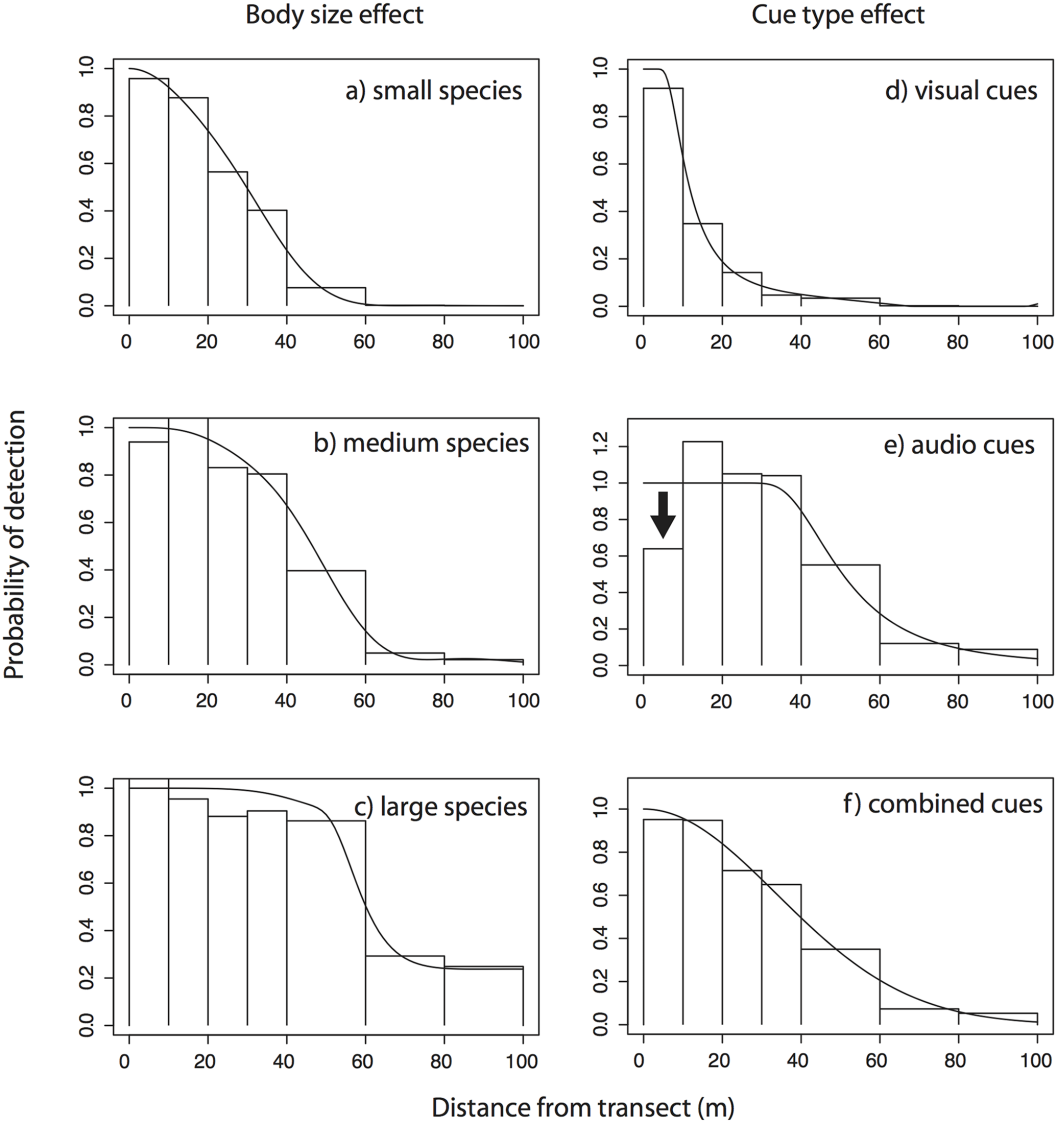
A comparison of the distance histograms and fitted detection functions between cues (right), and between body sizes (left). The arrow in (e) indicates the apparent deficit in audio detections at short distances resulting from the prioritisation of visual cues for accuracy of distance estimation during surveys.

**Fig 2:**
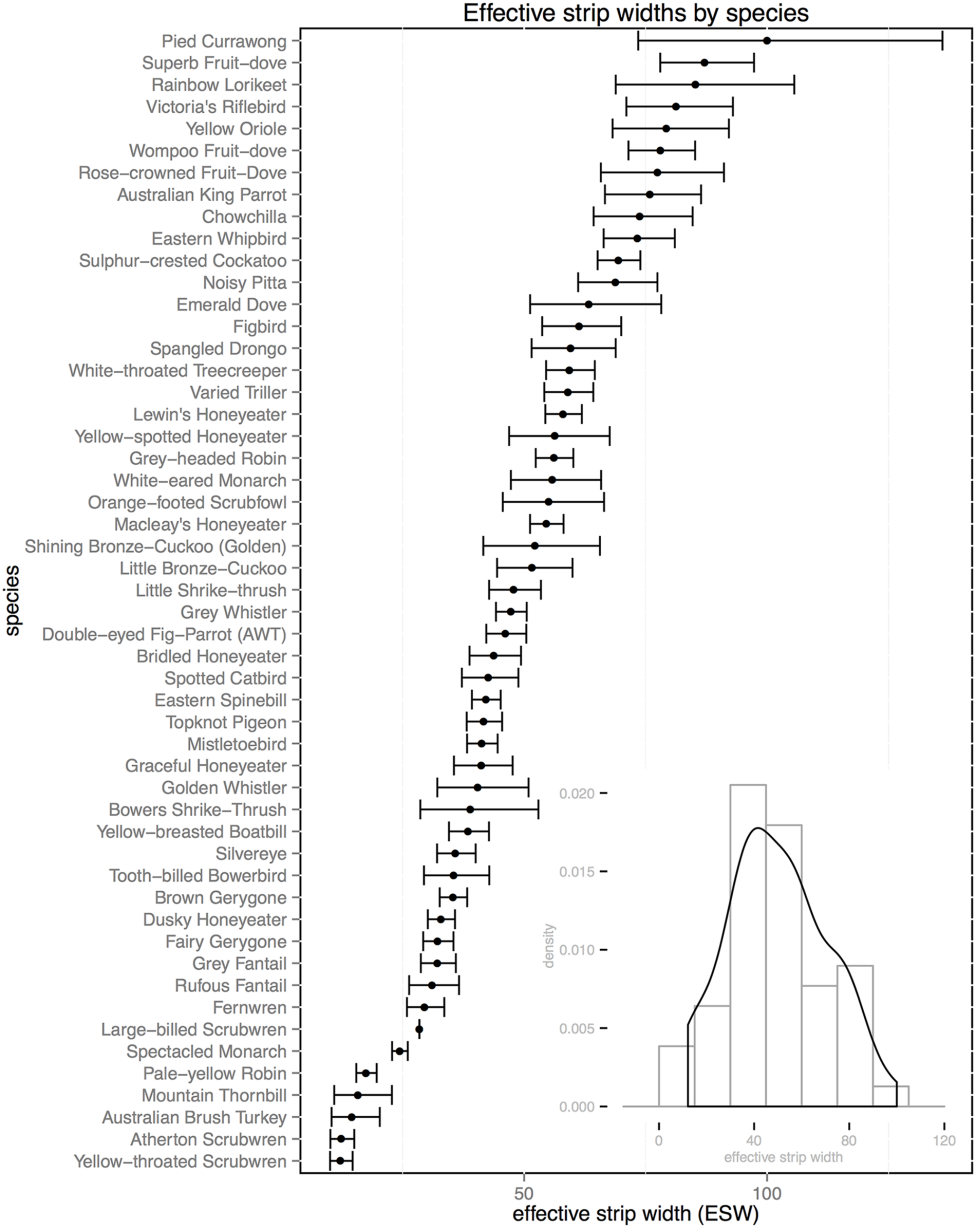
The distribution of estimated Effective Strip Width (ESW) 95% confidence intervals across all species examined, ranked in order of their estimated ESW from Distance analysis. An inset histogram shows the distribution of ESWs across all species, overlaid with with the probability density function for this distribution.

**Fig 3:**
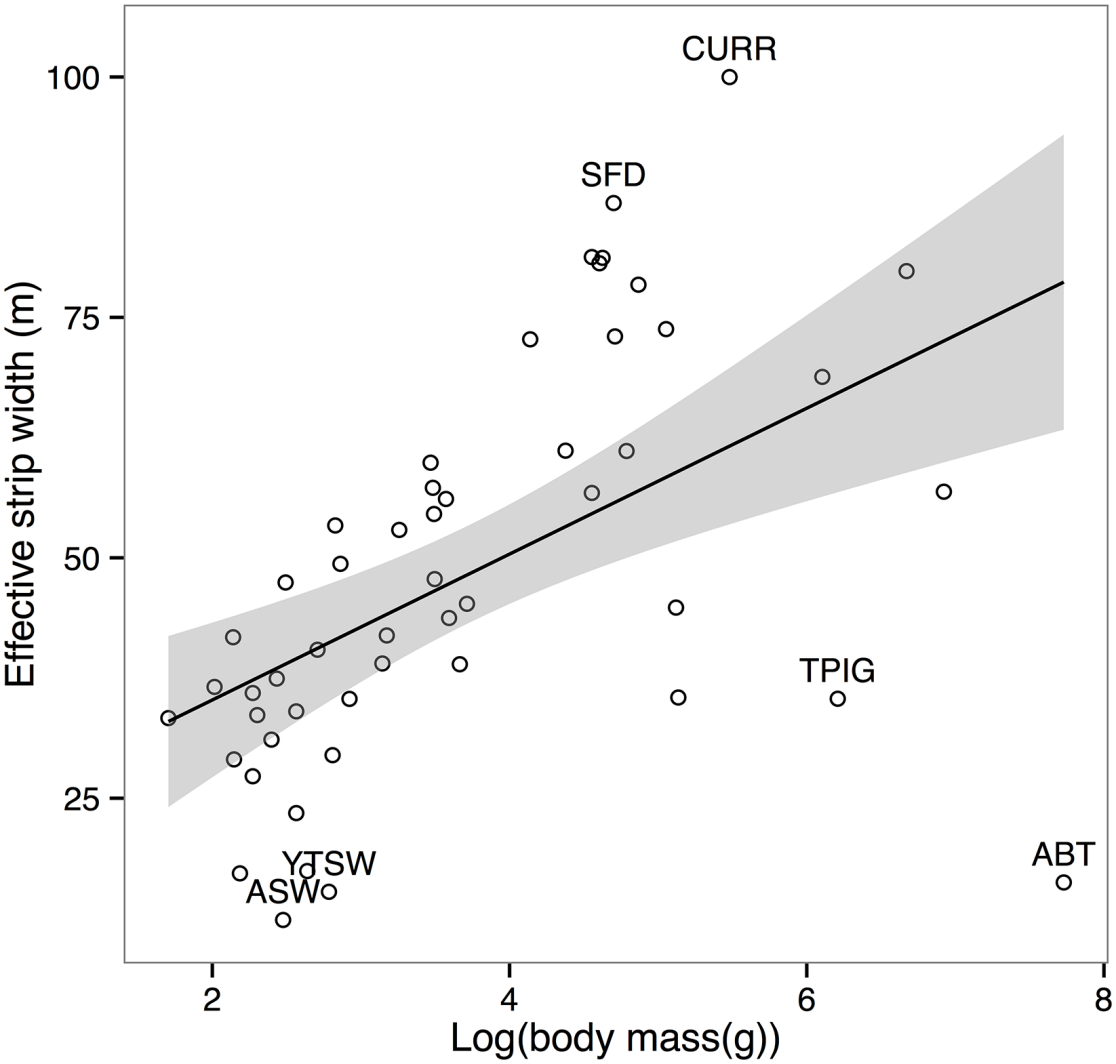
Estimated Effective Strip-widths plotted against log(body mass) for each of the 52 species compared here. See text for regression results. The shaded area shows 95% confidence intervals for the model including all species. Labelled points indicate species outlying to this trend: above the line two species with distinctive and penetrating calls CURR = (Pied Currawong (*Strepera graculina*), SFD = Superb Fruit-dove (*Ptilinopus superbus*). Below the line two species which rarely or only softly call (ABT = Australian Brush Turkey (*Alectura lathami*), TPIG = Topknot Pigeon (*Lopholaimus antarcticus*), one species more difficult to separate from congeners by call alone (ASW = Atherton Scrubwren (*Sericornis keri)*, and one species more likely to call when close to the observer ((per. obs.) YTSW = Yellow-throated Scrubwren (*Sericornis citreogularis*)).

Pooling across all species, surveys and sites, the best performing model in terms of AIC_c_ was one incorporating species as a factor covariate (Table 1), substantially better than the basic model without covariates. Correlated with species, body mass was also an important driver of differences in detectability, with smaller species (Fig 1a) showing a steady decay over shorter distances and no detections at greater distances; larger species (Fig 1c) showing the pronounced “shoulder” and long tail suggesting limited decay in detection probability over short to moderate distances, and medium-sized species showing an intermediate function (Fig 1b). In contrast, cluster size proved to have little influence on ESW in this study, both overall and on a per-species basis (see Figs C-D in S5 Fig, p-value = 0.25). Audio versus visual cue type was suggested as a more important covariate than body mass (Table 1) with ESWs tending to be much larger for audio records (Fig 1, right, mean difference = 21.64m, s.d. = 14.85m). As expected in audio-visual surveys [39], and even more so in closed forest, detection functions differed between audio (Fig 1d) and visual (Fig 1e) cue types. The apparent deficit of audio detections between 0 and 10 metres from the midline (arrow in Fig 1e) is attributable to the fact that, as is standard in such surveys, audio cues to nearby individuals where often tracked to their source. As described in the methods, this maximises the accuracy of both identification and distance estimation, but creates an apparent deficit of audio detections at small distances. Density estimated from audio data alone would be biased low as a result of this effect, but since combining audio and visual data (Fig 1f) removed any deficit of detections at small distances, we take the composite function to best represent the true shape across both cue types, and continue this treatment in all subsequent analyses. The importance of species as a covariate is driven by the pronounced variation in ESW evident between species (Fig 2), ranging from 11.62m to our truncation distance at 100m. A histogram of all ESWs (Fig 1, inset) showed also that while ESWs for most species cluster within 30 to 60 m (mean = 49.08m), variation was substantial (s.d. = 20.24m). Estimated ESW’s are given for each species in the supplementary material, S2 Table, along with their respective model adjustments, AIC values, estimated density and detection probability. Relative to the area surveyed for a hypothetical species whose ESW lies at the mean value for the assemblage, the shortest ESW equated to a reduction in ESW for that species from the assemblage mean of 75.6% (mean reduction 33%), while the largest equated to an increase of 100.6% (mean increase 35.6%). While the influence of size is apparent from the coarse categories shown in Fig 1a, 1b and 1c, and implied in the rankings of species along the y axis in Fig 2, plotting ESW against body mass as a continuous variable (Fig 3) illustrates this effect of size on detectability in more detail; ESW is significantly correlated with body mass (Adjusted R^2^: 0.2514, F: 17.79, p-value: 0.0001). Outliers to this pattern can be seen both above the trend line, e.g. Pied Currawong (*Strepera graculina*), Superb Fruit*-*dove (*Ptilinopus superbus*), and below it, e.g. Australian Brush Turkey (*Alectur*a *lathami*), Topknot Pigeon (*Lopholaimus* antarcticus), Yellow-throated Scrubwren (*Sericornis citreogularis*) and Atherton Scrubwren (*Sericornis keri*).

### Characteristics of surveys and habitat

**Fig 4:**
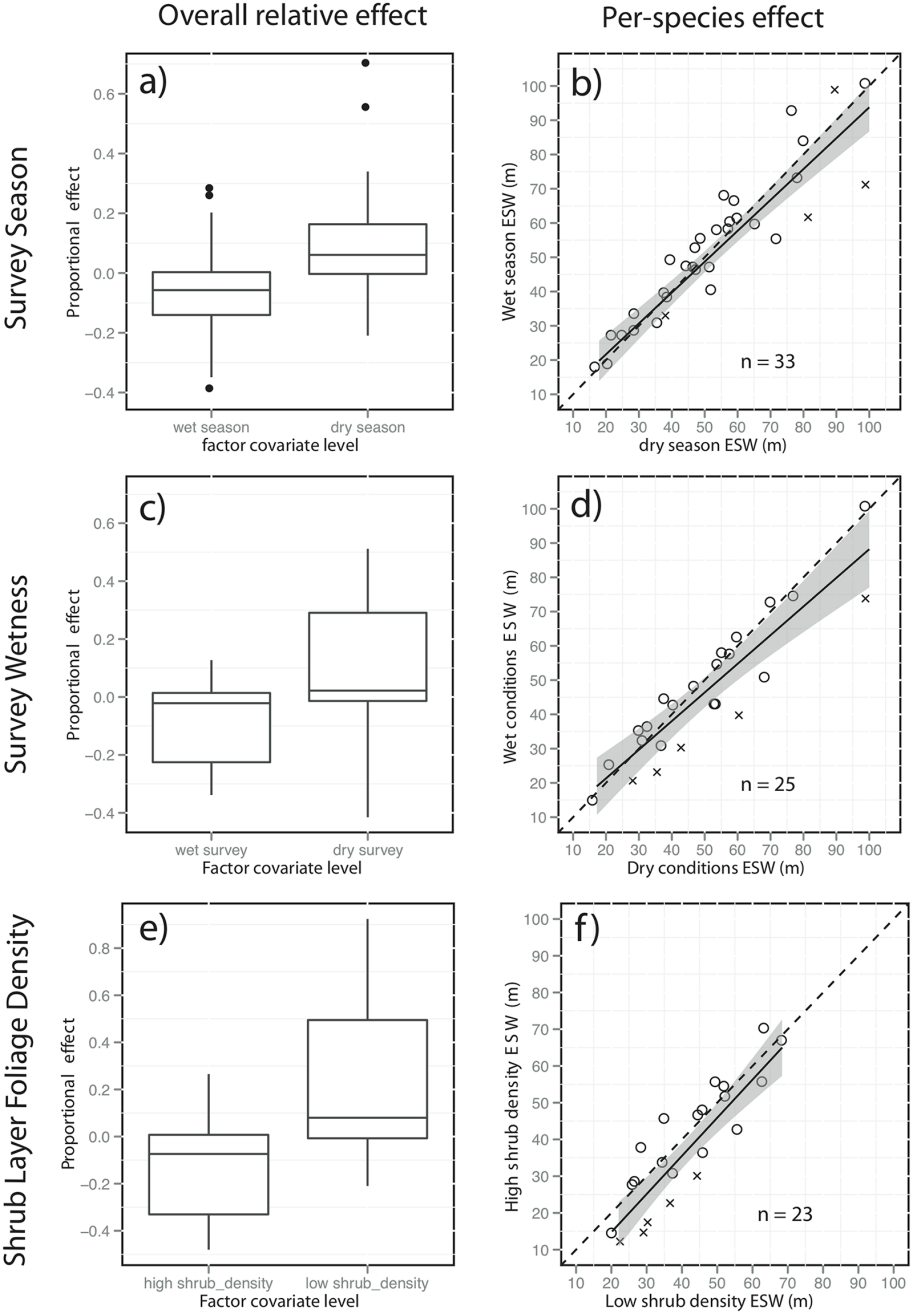
Left: Box-plots showing the relative effect of each covariate, expressed as the proportion of total ESW. Right: Biplots of each species showing the distribution of shifts in ESW. An “x” indicates those species with non-overlapping 95% confidence intervals (see text for details of species). Solid lines are linear regressions of the relationship, with 95% confidence intervals shaded.

The importance of species as a covariate, and the influence of body size on both the scale and shape of the detection function, reinforced the necessity of analysing covariate effects for each species separately. Overall, species-specific ESWs in summer surveys tended to be significantly shorter (Mann-Whitney U-test results in Table 2), than those during the winter (Fig 4a). Based on 95% confidence intervals, this seasonal effect on ESW was significant for four of the 33 species examined (Fig 4b): with lower wet-season ESW for (left to right) Silvereye (*Zosterops lateralis*), Wompoo Fruit-dove (*Ptilinopus magnficus*) and Superb Fruit-dove, and lower dry-season ESW for Victoria's Riflebird (*Ptiloris victoriae*). As an indication of the magnitude of the effect from a population estimation perspective, these species showed differences in the size of the effective strip width of + 34.8%, +25.3%, +40.6% and_-26.5% respectively. While we did not survey in heavy or persistent rain, “wet” surveys (when leaf litter is wet and foliage is dripping, a common feature in this and other rainforest field studies), had significantly shorter ESWs overall (Fig 4c), though the decrease was significant for only five of 25 species (Fig 4d): Large-billed Scrubwren (*Sericornis magnirostris)*, Rufous Fantail (*Rhipidura rufifrons*), Brown Gerygone (*Gerygone mouki*), Varied Triller (*Lalage leucomela*) and Superb Fruit-dove. These species showed differences in the size of the effective strip width of +25.7%, +33.8%, +28.6% +33.6% and +25.1 respectively. High shrub layer density was also associated with reduced ESW overall (Fig 4e), and had a significant negative effect for five of 30 species (Fig 4f): Yellow-throated Scrubwren, Grey Fantail, (*Rhipidura albiscapa)*, Silvereye, and Eastern Spinebill (*Acanthorhynchus tenuirostris*). These species showed differences in the size of the effective area surveyed of *+* 34.7%, +41.1%, +37.1% and 31.4% respectively. Interestingly, in each of these cases, the factor level associated with an increase in ESW was also associated with an increase in the proportion of detections from audio cues (data not shown). In contrast, lowland versus upland surveys had no overall significant difference in ESW, though ESW was significantly longer for two of 14 species in upland surveys (Figs A-B in S2 Fig, see captions for these and subsequent species details). Survey temperature relative to the site mean also had no overall significant influence, though ESW was significantly shorter for two of six species in warmer surveys (Figs C-D in S2 Fig). Transect placement on roads versus in forest had no overall significant influence on ESW, though significantly reduced ESW for one of 17 species, and increased it for two species (Figs E-F in S2 Fig). Neither high diversity of birds encountered on surveys (Figs A-B in S3 Fig, increase for three of 36 species, decrease for one) nor high abundance (Figs C-D in S3 Fig, increase for two of 35 species, decrease for three) appeared to have systematic influence on ESW. Habitat complexity (Figs E-F in S3 Fig), also showed no significant effect on ESW overall, and significant increased ESW for only one of 22 species. Wind during surveys had no overall significant effect on ESW at the intensities allowed by our sampling protocol, but significantly reduced ESW in one of 21 species, with "still" surveys showing greater ESW for 2 species (Figs A-B in S4 Fig). Noise level had no overall significant influence on ESW, though significantly reduced ESW for two of 15 species (Figs C-D in S4 Fig). Canopy density also did not affect ESW significantly overall, only slightly reducing ESW for one of 22 species (Figs E-F in S4 Fig). Rain during surveys at the intensities allowed by our sampling protocol had little systematic influence on ESW (Figs A-B in S5 Fig, one of 39 species), and nor did group size (Figs C-D in S5 Fig) with increase in ESW for detections of single individuals over clusters for only one of 11 species.

**Table 2:** Results of overall and per-species analyses of the effects of each factor covariate on ESW.

	Model factor	Mean ESW Difference	Influence on ESW	Mann-Whitney p-value	Proportion of significant differences	N
1	Elevation	-9.08	Negative	0.50	0.14	14.00
2	Temperature	1.23	Positive	0.19	0.17	17.00
3	Route	3.59	Positive	0.12	0.18	6.00
4	**Wetness**	**4.14**	**Lower when wet**	**0.03**	**0.20**	**25.00**
5	Bird diversity	0.17	Positive	0.46	0.06	36.00
6	Bird abundance	0.97	Positive	0.257	0.14	37.00
7	Complexity	3.21	Positive	0.10	0.05	20.00
8	Wind	2.71	Positive	0.29	0.14	21.00
9	Noise	2.79	Positive	0.17	0.13	15.00
10	Canopy density	2.58	Positive	0.10	0.05	22.00
11	**Shrub density**	**4.48**	**Positive**	**0.01**	**0.17**	**23.00**
12	**Season**	**-2.13**	**Lower in Summer**	**0.05**	**0.12**	**33.00**
13	Rain	-1.16	Negative	0.25	0.03	39.00
14	Cluster size	8.19	Positive	0.25	0.09	11.00

Shown are significant covariate effects on Effective Strip Widths, based on Mann-Whitney U tests. The proportion of tested species showing non-overlapping 95% confidence are indicated in bold.

## Discussion

### Characteristics of detected objects

We show that interspecific differences in detectability with distance are critical for density correction in our region-wide and multi-species surveys of montane tropical rainforest birds, potentially resulting in substantial over- and under-estimates of survey area if a fixed width is used, and hence corresponding under- or over-estimates of density respectively. These differences were correlated to body size, and affected both the shape and scale of detection functions, highlighting the importance of species-specific corrections for detectability. This also suggests a primary goal of sampling designs in these conditions should be achieving adequate replication of distance samples for fitting individual detection functions to taxa of interest. Using both audio and visual cues helps by maximising the available detections in rainforest, but as expected, cue types showed markedly different detection functions in this study. In such cases Buckland et al. [6] recommend analyzing data from calls and sightings separately, but we prioritised sightings for their increased confidence in identification and distance estimation, resulting in an apparent deficit of aural detections at and near the transect midline when these are analysed separately. Combining audio and visual cues, however, gave both a plausible detection function with an adequate shoulder, and sufficient data for ~70% of species, justifying pooling of data for both cue types. We suggest our pooling approach as a practical alternative to the more complex protocol of noting when birds are both seen *and* heard (but see, e.g. [43]).

Given the wide variation in morphology, call characteristics and behaviour among birds, it is unsurprising that after cue type, species was the primary influence on detection probability. Both the magnitude of the observed variation and the fact that it is roughly continuous makes it difficult to justify using the mean value of ESW to characterise the area surveyed for all species in this community: using the mean here would result in an average ~30% overestimate of ESW, and hence underestimate of density, across all species. This inflates to as much as ~310% percent ESW overestimate (or density underestimate) for some smaller and less detectable species, or conversely a ~50% ESW underestimate (or density overestimate) for larger-bodied species. However, collecting the necessary species-specific data to account for these differences may be a costly process in the diverse bird communities of tropical rainforest, where some species are rarely encountered. As an alternative, Alldredge et al. [55] recommend borrowing information about detectability from species with similar maximum detection distance. For these problematic rare species, Mackenzie et al. [56] also suggested sharing data between those with similar visibility, activity patterns, size and behaviour. Some field studies have considered this *a priori* [46,57], using subjective assessments of habitat use or activity level, assumed to influence detectability. Our results indicate that body size is a potentially important grouping covariate for sharing detectability information, with the advantage of being both objective and readily available in the literature. Importantly, this is despite the fact that here about 80% of detections were aural. We suggest this may be due to correlation between body size and call characteristics, since larger species may call more loudly [58] and at lower frequencies than smaller species, resulting in calls which attenuate less rapidly [40], and remain distinguishable at greater distances from the observer (A.A. pers. obs). Differences in calling behaviour, including timing (both annual and diel), frequency, volume, and reactions to observers, however, may drive outliers from this relationship. Here, outliers tended to be species with unusual call characteristics, either calling loudly and often (species well above the trend line in (e.g. Pied Currawong (*Strepera graculina*), Superb Fruit-dove (*Ptilinopus superbus*)), or quietly and/or infrequently (species well below the trend line, e.g. Australian Brush Turkey (*Alectura lathami*), Topknot Pigeon (*Lopholaimus antarcticus*)). Additionally, ESW is lower than expected from body size for one species that is more difficult to separate from congeners by call alone (Atherton Scrubwren (*Sericornis keri*), and for one which may be more likely to call when close to the observer ((A.A. pers. obs.) Yellow-throated Scrubwren (*Sericornis citreogularis*)). We suggest that a detailed study of grouping covariates for detectability should therefore also include a treatment of species’ call characteristics and behaviour.

### Characteristics of surveys

Time of season may be an important influence on density estimation in bird surveys [59,60]. All three species which showed here an increase in ESW during the dry season are canopy frugivores, two of which (Wompoo Fruit-dove and Superb Fruit-dove) were noted by Crome [61] to have a peak in calling activity during the dry season when they breed, synchronous with maximum fruit availability. In contrast, Victoria's Riflebird, which displays and breeds from September to January [47], showed higher ESW during the wet season. This suggests that some seasonal differences between ESW may stem from variation in rates of audio cue production, and hence the proportion of audio detections. While surveying in both seasons mitigates any bias in overall density estimates, the inclusion of a season covariate for such species would be recommended where the spread of sampling is not sufficient to capture temporal variation in detectability.

Here as in [20] sampling was not conducted on excessively rainy or windy days, or in periods of substantial background noise, and the lack of systematic influence of these covariates on ESW suggests that our protocol eliminates most of the effects of poor weather conditions, which may otherwise influence detectability [28]. The lack of observer “swamping” in this study, even at high diversity and abundance, would also suggest only a weak effect, and/or sufficient observer training and experience to overcome it. Wetness however remained a significant influence on ESW for some species. Canopy-drip in rainforest is common both after rain and in cloudy conditions at high elevations, and we suggest that in addition to a decrease in bird activity and hence availability for detection, increased background noise from dripping foliage may also decrease detection distances. Comparisons of density estimates between sites where wetness varies systematically, for example across large climatic gradients, may therefore be biased. In these cases, care should therefore be taken to ensure sampling is either a) conducted extensively in optimal conditions, or b) that relevant weather measures are included as covariates.

### Characteristics of habitat

In contrast to surveys in open habitats in Australia’s tropical north (e.g. [62]) a reduced role for the attenuation of visual cues could be expected in audio-cue dominated surveys characteristic of rainforest [14]. Nonetheless, shrub-layer foliage density had a significant negative influence on ESW overall, and a significant negative effect for five species. This effect was concentrated over shorter distances (and hence disproportionately influenced detection of smaller species), and translated to a mean decrease of 40% in ESW in sites with dense understory. If uncorrected, relative abundance estimates may therefore be biased where shrub-layer density varies substantially. While sighting birds in dense foliage is difficult, this effect may also involve increased attenuation of call sounds by reverberation and reflection [63]. Regardless of its cause, this result suggests that where site-specific density estimates are not made directly, a shrub layer covariate should be included to correct for detectability differences between sites with substantial vegetation structural differences in the understorey. Overall, covariates of detectability in the rainforest bird surveys we analyse therefore appear to influence detectability through two interacting pathways: firstly through rates of cue production, and hence differences in the proportion of visual versus audio cues, and secondly via attenuation of cues.

Importantly however, we note that cue production will also have an influence on availability: the probability of individuals present producing a detectable cue. As we have shown, availability may vary between species, season, or under different weather or site conditions. Birds may not call when observers are within range, or may not be heard amongst other sounds [14,64]. An observer's field of view on the forest floor is often limited, and birds close to the transect may be 40 m above in the canopy, or obscured in dense foliage. In such cases where availability is less than 1, a key assumption of distance sampling is not met: that probability of detection at g(0) (the transect midline) is also equal to 1 [6,11].]. In our study, combining audio and visual cues and surveying at dawn maximises availability, but potential remains for individuals even at the transect midline to go undetected, especially in tall forests. Despite the fact that this problem may be common [65], especially in the tropics [14], relatively few studies treat availability separately. We suggest that quantifying the prevalence and magnitude of underestimates in forest bird densities that result from availability being less than 1 should be a priority in future studies. Gale et. al. [14] suggest territory mapping as a possible, albeit labour-intensive solution. Another approach may be to compare conventional survey results with those made by accessing the canopy, which may yield detections of individuals and species not possible otherwise [66]. A longer sample duration may also serve to increase availability for species that call infrequently or are canopy dwelling, but may come at cost of upward bias from double counting. Pooling consecutive surveys for these species may also be an alternative.

### Other limitations and sources of error

Error in density estimation may also arise from inaccuracy in the measurement of distances [41,42]. Since distances to audio cues are often estimated, this source of error may be more important in audio-dominated forest bird surveys [30,43]. Error rates in this localization process also increase with distance [67] and with increasing call frequency (pitch) [40]. Here we attempted to reduce the impact of estimation error by increasing bin sizes with increasing distance but we recognise that an unknown error component remains due to subjective assessment of audio cue distances. Unfortunately, the paucity of sightings in rainforest surveys limits the scope for practical alternatives: if audio cures are excluded, sufficient data will be difficult to accumulate for all but the most commonly-sighted species, and visual detectability decay may be too steep to accurately fit a detection function. In some settings, calibration experiments could be used to characterise the error structure [68]. Newly developed techniques such as “acoustic spatially explicit capture-recapture” might prove useful [69]. Additional methods such as radio-telemetry have been suggested [65], but may be impractical and uneconomical to apply over the spatial and temporal scales required. However, we strongly suggest that measures of the error structure of distance estimation to audio cues should be incorporated into forest bird surveys in future. One promising avenue may be the use of passive acoustic arrays [70], and some advances have already been made for single species systems [71,72], suggesting scaling to multiple-species surveys to be the next challenge. If information about the measurement error is available, it can be readily incorporated into the Distance analysis framework [68,73].

Bird movements relative to the observer can also result in bias if they are more rapid than about half the observer’s rate of movement [10]. This can occur as a result of either: 1: accumulation of individuals rather than a “snapshot” count [44]; 2 double counting; or 3: actual departure from uniform density with distance from the midline, due to birds avoiding or approaching the observer [17]. Moving more rapidly than the objects of the survey is one method for avoiding the first source of error [17]. In our case, a key goal of the monitoring program is to measure diversity at sites. Bird diversity is high in the AWT (as many as 35 species present on a single transect), so that we found a slower survey rate necessary to detect and identify all the individuals and species present. As in many montane tropical forest environments, terrain and vegetation also present challenges. Our strategy, a survey rate of 5 m/min, is slightly slower than other temperate surveys (e.g [38,48,74–80], mean = 12.19m/min), but not unusual among tropical bird surveys (e.g [14,20,81–84] mean 7.73 m/min), where high diversity and complex vegetation and/or terrain may dictate a slower pace [14] than in temperate surveys. Our “look-forward” survey method, suggested in [45], further minimises bias due to avoidance or attraction movements by locating individuals in a moving window ahead of the observer before they react to our presence. This method can, in theory, further increase the period in which detected individuals have time to either 1: move and be replaced by new individuals which are added to the count or 2: move ahead to a new location and be spuriously double counted, so that care must thus be taken to minimise these sources of bias. We employ a compromise approach that uses a short survey window ahead of the observer, in fact dictated by the short visual range in closed forest (approximately 20–30 metres, similar to the limit recommended by [85]), and by the maximum distance from the observer that can be reasonably estimated for audio cues (approximately 60 metres [42]). We further lower the risk of double counting and accumulation by recording data in 6 sub-segments of 5 minutes duration each. This allows the observer to build a “spatio-temporal map” of the individuals present. A glance at the data-sheet gives quick overview of the time (and hence distance along the midline) since a species was last recorded, allowing an informed decision as to whether or not a detection constitutes a new individual. We are also conservative in this regard, tending to attribute new cues to previously detected individuals whenever there is some doubt.

In the case of bird movements leading to non-uniform density with respect to the transect, an advantage of line transect surveys over point counts is that birds on the midline can more readily be counted either before they are disturbed, or recorded at the point from which they flush, reducing this bias [4]. Shy species, however, may still be affected if their movements occur outside the look-ahead window. Here examination of the compound detection functions for each species shows little deficit at the midline, suggesting this problem is limited in the current study, but further comparisons with data from surveys using a longer look-ahead windows, or from full census sampling, would be useful for quantifying variation between species. Fortunately, distance-corrected transects surveys still compare well to alternative methods [9] and even in complex rainforest environments, line-transect distance sampling surveys return robust and relatively unbiased estimates when measured against other approaches [14,18]. Careful and systematic sampling by well-trained and experienced observers may thus remain remain the most cost-effective solution for reducing error rates in general [28], and for sound cue localization in particular [42,48]. Where the deviations from critical assumptions are suspected to be large, more intensive or specifically targeted methods could be applied, such as double-observer sampling (e.g. [86]).

While cluster size had little influence on detection probability in this study, single-species clusters made up a relatively small proportion of our records. We were conservative in assigning individuals to clusters, so that individuals in loosely defined groups were treated as single individuals. We also did not attempt to collect data to distinguish detections of members of mixed-species flocks. Mixed flocking has been noted frequently in Australian woodland birds [47], and though it may be a common phenomenon in the region [87], there is little in the literature for rainforest species,. Since detectability of cryptic members may increase in flocks containing more obvious species, mixed flocking thus remains a potential source of bias that warrants exploration. Lastly, while distance sampling assumes that transects are placed at random in the landscape, access in tropical forests is a challenge, and may depend on roads and tracks which are not randomly placed. This can influence estimation where density also varies with distance from roads [35]. For example, some species may be attracted to roads, while are repelled [88,89]. A secondary effect may arise from influences on habitat structure [90]; in rainforests, increased light availability along roads may alter the structure of vegetation [91] and hence effect the attenuation of survey cues. Here surveys were conducted along roads too infrequently to explore their effects in detail, but some species show indication of road influence on density. The most common effect appears to be attraction, largely restricted to understorey insectivores (Fig F in S2 Fig), perhaps due to microclimate effects on insect activity. Where surveys commonly use roads, or do so systematically with respect to the overall sampling design, this effect could bias density estimates for the effected species. We suggest that surveys on roads be avoided in general, but where unavoidable, information on habitat structure gradients could be collected and included in the Distance analysis [35].

### Suggested protocols for rainforest bird surveys, and further work

Based on the analyses presented here, we can outline key features of rainforest bird sampling protocols that should minimise the influence of important sources of bias. Firstly, species, habitat structure and survey effects (including season and wetness) suggest distance sampling will substantially improve estimates of density in rainforests, provided that 1: sampling is sufficient to estimate density for each species of interest, 2: information about habitat structure at least in the shrub layer is collected, 3: surveys are conducted under optimum conditions (including seasonal sampling), but that information about environmental wetness is recorded as a minimum requirement in rainforest surveys. We also suggest incorporating some effort to evaluate 1: availability for detection (e.g. call rates), and 2: distance estimation error. This might be done in a calibration subset of surveys where calling individuals are monitored, and their position is more accurately mapped (see e.g., [42]). It is also important to note that in an assemblage-wide study, species differences may be the more significant contributors to bias in density estimates, particularly were assemblage composition changes across the study region. Studies concerned with differences between sites or time periods within a single species however, such as monitoring of population changes, will likely need to counter the bias introduced by site and survey differences instead.

The results presented here also indicate approaches that may be useful where data are too sparse for Distance analysis, or where formal distance sampling may not be practicable in all surveys (as in large-scale or volunteer-based surveys). In the absence of ESW estimates for important covariates (including species), to be comparable surveys should at minimum: 1; pool data across sites with regard to averaging out variation due to habitat structure, especially in the shrub layer, 2: pool data across surveys with regard to averaging out variation due to survey wetness and season, and 3: share detectability information across species data based on shared characteristics (e.g., body size). Lastly, the importance of species as a covariate shown here also suggests a data-driven approach to modelling abundance of rare species: a challenge often encountered in distance sampling, and indeed whenever it is important to account for detectability in survey data. To guide the sharing of detection data for rare species, or those for which distance data are lacking, we suggest that a useful starting point will be a proper consideration of the influence of body size and call characteristics, followed by examination of other covariates related to species behaviour or morphology. In combination with distance sampling and the minimum survey requirements we suggest here, such an approach may prove especially useful in addressing some of the challenges faced in the urgent task of monitoring bird populations in tropical forest environments.

## Supporting Information

S1 Fig Schematic of the audio-visual bird survey method with distance sampling. Transects were walked for 30 minutes, and the distances to all birds seen or heard were estimated or measured directly where possible. 1) Distances to birds seen close to the transect were measured with a laser rangefinder. 2) Birds on the transect midline were recorded at zero meters before they move to avoid the observer. 3) Distances to birds calling from concealment within 50m of the transect were estimated, later binned to 10m intervals. 4) and 5) A single distance to groups of birds was measured or estimated to the group centre, and number of individuals counted or estimated. 6) Birds heard calling at distances estimated to be greater than 100m were excluded from later analyses, as distance estimation becomes unreliable at larger distances. 7) Distances to birds estimated to be calling from between 50m and 100m were later binned in 20m categories. 8) Distances to birds heard calling from close to the transect well ahead of the observer were estimated accordingly, and later confirmed visually were possible. Estimated heights to seen individuals were also recorded.
(PDF)

S2 Fig A comparison of the relative effect of elevation, temperature and route covariates on Effective Strip Width (ESW). Box-plots on left (Figs A,C,E) show the median, 25th and 75th quantile of the range of ESW relative differences between treatments, expressed as the proportion of each species’ total ESW. Biplots (Figs B, D, F) on the right show the distribution among species of shifts in ESW associated with each covariate. N values refer to the number of species compared and an “x” marks those with non-overlapping 95% confidence intervals. For elevation (Fig B) these are (left to right within plots): Large-billed Scrubwren (*Sericornis magnirostris*) and Spotted Catbird (*Ailuroedus melanotis*), for temperature (Fig D) Mistletoebird (*Dicaeum hirundinaceum*), and for survey route (Fig F), Large-billed Scrubwren, Spotted Catbird and Superb Fruit-dove (*Ptilinopus superbus*). Solid lines indicate a simple linear regression of the relationship, upper and lower 95% confidence intervals are shaded, relative to zero difference (dashed line).
(PDF)

S3 Fig A comparison of the relative effect of bird diversity, bird abundance and habitat complexity covariates on Effective Strip Width (ESW). Box-plots on left (Figs A,C,E) show the median, 25th and 75th quantile of the range of ESW relative differences between treatments, expressed as the proportion of each species’ total ESW. Biplots (Figs B, D, F) on the right show the distribution among species of shifts in ESW associated with each covariate. N values refer to the number of species compared and an “x” marks those with non-overlapping 95% confidence intervals. For diversity (Fig B) these are (left to right within plots): Silvereye (Zosterops lateralis), Eastern Spinebill (Acanthoryhnchus tenuirostris) and Victoria’s Riflebird (Ptiloris victoriae), for abundance (Fig D) Yellow-throated Scrubwren (Sericornis magnirostris), Silvereye, Mistletoebird (Dicaeum hirundinaceum), Superb Fruit-dove (Ptilinopus superbus) and Victoria’s Riflebird and for habitat complexity (Fig F) Superb Fruit-dove. Solid lines indicate a simple linear regression of the relationship, with upper and lower 95% confidence intervals shaded, relative to zero difference (dashed line).
(PDF)

S4 Fig A comparison of the relative effect of wind, noise and canopy density covariates on Effective Strip Width (ESW). Box-plots on left (Figs A,C,E) show the median, 25th and 75th quantile of the range of ESW relative differences between treatments, expressed as the proportion of each species’ total ESW. Biplots (Figs B, D, F) on the right show the distribution among species of shifts in ESW associated with each covariate. N values refer to the number of species compared and an “x” marks those with non-overlapping 95% confidence intervals. For wind (Fig B) these are (left to right within plots): Mountain Thornbill (*Acanthiza katherina*), Bridled Honeyeater (*Lichenostomus frenatus*) and Lewin’s Honeyeater (*Meliphaga lewinii*), for noise (Fig D) Little Shrike-thrush (*Coluricincla megarhyncha*) and Grey Whistler (*Pachycephala simplex*) and for canopy density (Fig F) Golden Whistler (*Pachycephala pectoralis*). Solid lines indicate a simple linear regression of the relationship, with upper and lower 95% confidence intervals shaded, relative to zero difference (dashed line).
(PDF)

S5 Fig A comparison of the relative effect of rain and cluster size covariates on Effective Strip Width (ESW). Box-plots on left (Figs A, C) show the median, 25th and 75th quantile of the range of ESW relative differences between treatments, expressed as the proportion of each species’ total ESW. Biplots on the right (Figs B, D) show the distribution among species of shifts in ESW associated with each covariate. N values refer to the number of species compared, an “x” marks those with non-overlapping 95% confidence intervals. For rain (Fig B) these are Mistletoebird (*Dicaeum hirundinaceum*), and for cluster size (Fig D) Silvereye (*Zosterops lateralis*).
(PDF)

S1 Table Description of the scoring system used to classify survey wind, rain, wetness and noise conditions into relative scores. These scores were used firstly to guide decisions when to abandon surveys, and secondarily as covariates in the detectability analyses presented here.
(PDF)

S2 Table Estimates of ESW (effective strip width) by species from Distance sampling and analysis in Australian Wet Tropics (AWT). AIC_c_ refers to AIC values corrected for small sample size. Density values refer to an estimate per hectare based on the mean counts across all surveys. Model abbreviations are as follows: hn = Half-normal, hr = Hazard rate, uni = Uniform, (see methods, and Thomas et al. (2010) for a detailed description).
(PDF)
