# Supplementary material for: Detectability in Audio-Visual Surveys of Tropical Rainforest Birds: The Influence of Species, Weather and Habitat Characteristics
Source: PLoS One. 2015 Jun 25;10(6):e0128464. doi: 10.1371/journal.pone.0128464 (PMC4482497; doi:10.1371/journal.pone.0128464)
Supplement: S1 Fig — Transects were walked for 30 minutes, and the distances to all birds seen or heard were estimated or measured directly where possible. 1) Distances to birds seen close to the transect were measured with a laser rangefinder. 2) Birds on the transect midline were recorded at zero meters before they move to avoid the observer. 3) Distances to birds calling from concealment within 50m of the transect were estimated, later binned to 10m intervals. 4) and 5) A single distance to groups of birds was measured or estimated to the group centre, and number of individuals counted or estimated. 6) Birds heard calling at distances estimated to be greater than 100m were excluded from later analyses, as distance estimation becomes unreliable at larger distances. 7) Distances to birds estimated to be calling from between 50m and 100m were later binned in 20m categories. 8) Distances to birds heard calling from close to the transect well ahead of the observer were estimated accordingly, and later confirmed visually were possible. Estimated heights to seen individuals were also recorded. (PDF) [file pone.0128464.s001.pdf]

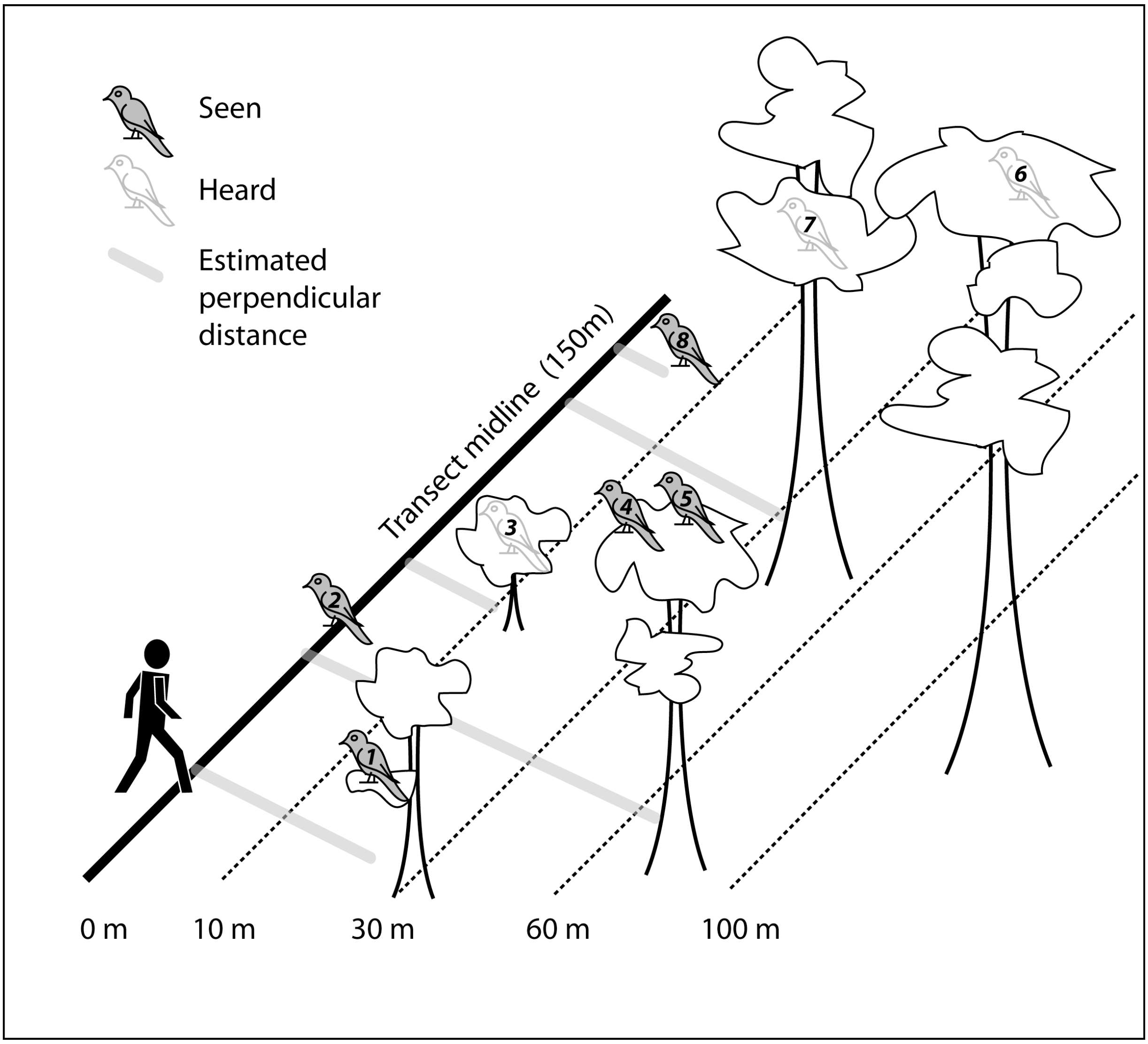

S1 Fig. **Schematic of the audio-visual bird survey method with distance sampling**. Transects were walked for 30 minutes, and the distances to all birds seen or heard were estimated or measured directly where possible. 1) Distances to birds seen close to the transect were measured with a laser rangefinder. 2) Birds on the transect midline were recorded at zero metres before they move to avoid the observer. 3) Distances to birds calling from concealment within 50m of the transect were estimated, later binned to 10m intervals. 4) and 5) A single distance to groups of birds was measured or estimated to the group centre, and number of individuals counted or estimated. 6) Birds heard calling at distances estimated to be greater than 100m were excluded from later analyses, as distance estimation becomes unreliable at larger distances. 7) Distances to birds estimated to be calling from between 50m and 100m were later binned in 20m categories. 8) Distances to birds heard calling from close to the transect well ahead of the observer were estimated accordingly, and later confirmed visually were possible. Estimated heights to seen individuals were also recorded.
