# Supplementary material for: Detectability in Audio-Visual Surveys of Tropical Rainforest Birds: The Influence of Species, Weather and Habitat Characteristics
Source: PLoS One. 2015 Jun 25;10(6):e0128464. doi: 10.1371/journal.pone.0128464 (PMC4482497; doi:10.1371/journal.pone.0128464)
Supplement: S2 Fig — Box-plots on left (Figs A,C,E) show the median, 25th and 75th quantile of the range of ESW relative differences between treatments, expressed as the proportion of each species’ total ESW. Biplots (Figs B, D, F) on the right show the distribution among species of shifts in ESW associated with each covariate. N values refer to the number of species compared and an “x” marks those with non-overlapping 95% confidence intervals. For elevation (Fig B) these are (left to right within plots): Large-billed Scrubwren (Sericornis magnirostris) and Spotted Catbird (Ailuroedus melanotis), for temperature (Fig D) Mistletoebird (Dicaeum hirundinaceum), and for survey route (Fig F), Large-billed Scrubwren, Spotted Catbird and Superb Fruit-dove (Ptilinopus superbus). Solid lines indicate a simple linear regression of the relationship, upper and lower 95% confidence intervals are shaded, relative to zero difference (dashed line). (PDF) [file pone.0128464.s002.pdf]

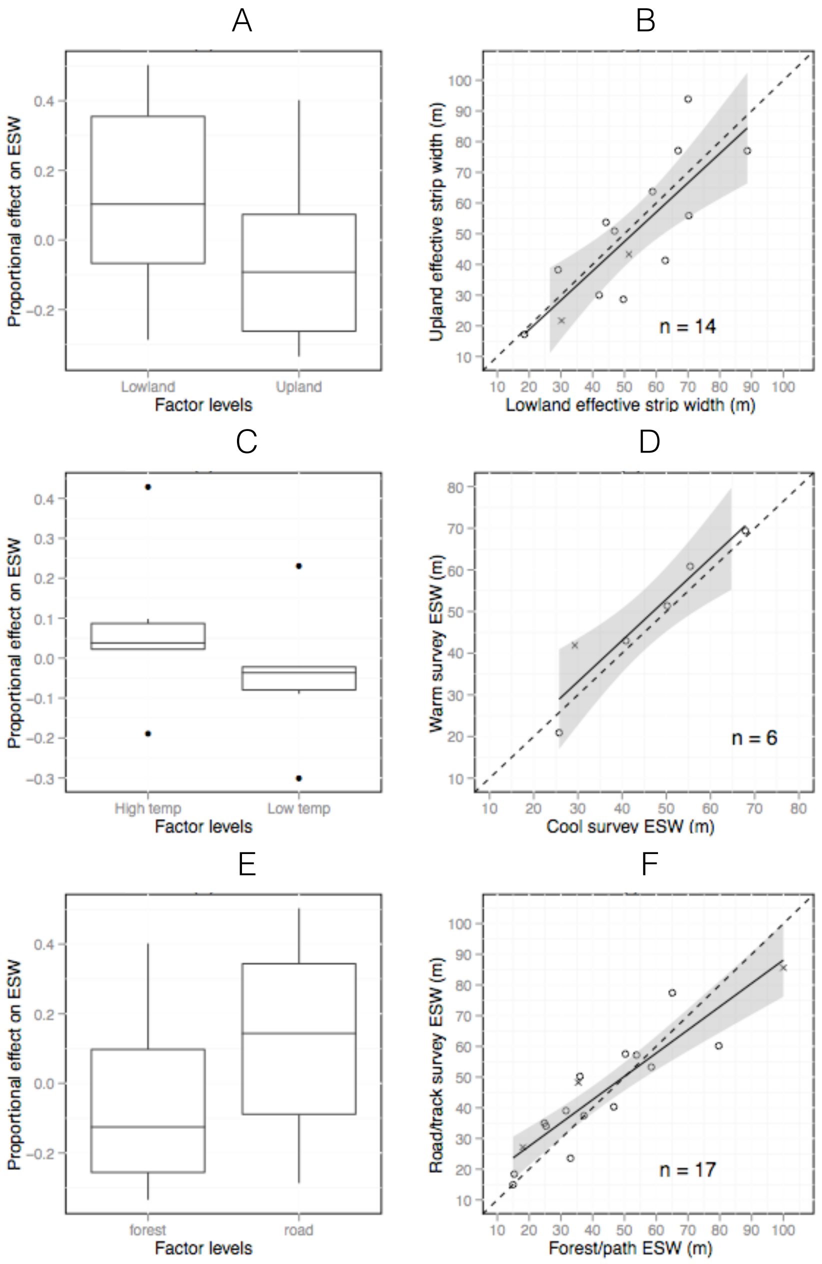

S2 Figs. A comparison of the relative effect of elevation, temperature and route covariates on Effective Strip Width (ESW). Box-plots on left (Figs. A,C,E) show the median, 25th and 75th quantile of the range of ESW relative differences between treatments, expressed as the proportion of each species' total ESW. Biplots (Figs. B, D, F) on the right show the distribution among species of shifts in ESW associated with each covariate. N values refer to the number of species compared and an "x" marks those with non-overlapping 95% confidence intervals. For elevation (Fig. B) these are (left to right within plots): Large-billed Scrubwren (*Sericornis magnirostris*) and Spotted Catbird (*Ailuroedus melanotis*), for temperature (Fig. D) Mistletoebird (*Dicaeum hirundinaceum*), and for survey route (Fig. F), Large-billed Scrubwren, Spotted Catbird and Superb Fruit-dove (*Ptilinopus superbus*). Solid lines indicate a simple linear regression of the relationship, upper and lower 95% confidence intervals are shaded, relative to zero difference (dashed line).
