# Supplementary material for: Detectability in Audio-Visual Surveys of Tropical Rainforest Birds: The Influence of Species, Weather and Habitat Characteristics
Source: PLoS One. 2015 Jun 25;10(6):e0128464. doi: 10.1371/journal.pone.0128464 (PMC4482497; doi:10.1371/journal.pone.0128464)
Supplement: S4 Fig — Box-plots on left (Figs A,C,E) show the median, 25th and 75th quantile of the range of ESW relative differences between treatments, expressed as the proportion of each species’ total ESW. Biplots (Figs B, D, F) on the right show the distribution among species of shifts in ESW associated with each covariate. N values refer to the number of species compared and an “x” marks those with non-overlapping 95% confidence intervals. For wind (Fig B) these are (left to right within plots): Mountain Thornbill (Acanthiza katherina), Bridled Honeyeater (Lichenostomus frenatus) and Lewin’s Honeyeater (Meliphaga lewinii), for noise (Fig D) Little Shrike-thrush (Coluricincla megarhyncha) and Grey Whistler (Pachycephala simplex) and for canopy density (Fig F) Golden Whistler (Pachycephala pectoralis). Solid lines indicate a simple linear regression of the relationship, with upper and lower 95% confidence intervals shaded, relative to zero difference (dashed line). (PDF) [file pone.0128464.s004.pdf]

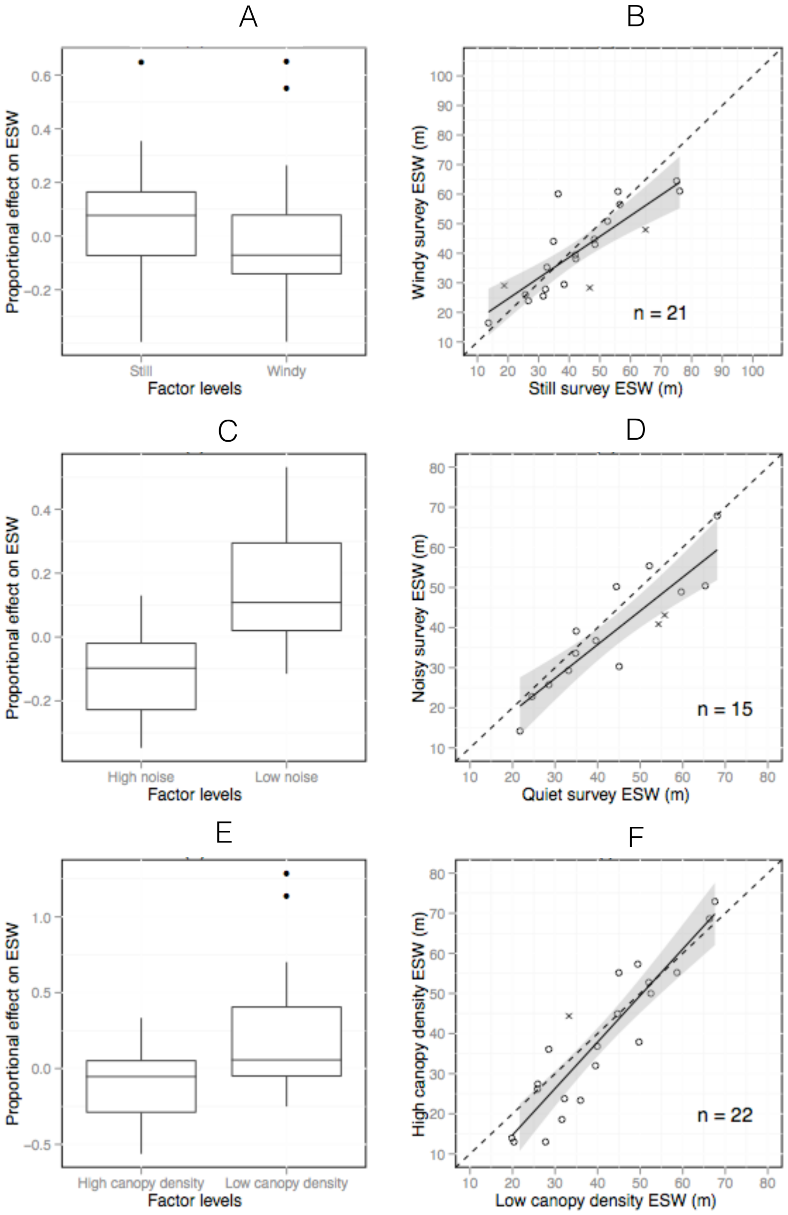

S4 Figs. A comparison of the relative effect of wind, noise and canopy density covariates on Effective Strip Width (ESW). Box-plots on left (Figs. A,C,E) show the median, 25th and 75th quantile of the range of ESW relative differences between treatments, expressed as the proportion of each species' total ESW. Biplots (Figs. B, D, F) on the right show the distribution among species of shifts in ESW associated with each covariate. N values refer to the number of species compared and an "x" marks those with non-overlapping 95% confidence intervals. For wind (Fig. B) these are (left to right within plots): Mountain Thornbill (*Acanthiza katherina*), Bridled Honeyeater (*Lichenostomus frenatus*) and Lewin's Honeyeater (*Meliphaga lewinii*), for noise (Fig. D) Little Shrike-thrush (*Colluricincla megarhyncha*) and Grey Whistler (*Pachycephala simplex*) and for canopy density (Fig. F) Golden Whistler (*Pachycephala pectoralis*). Solid lines indicate a simple linear regression of the relationship, with upper and lower 95% confidence intervals shaded, relative to zero difference (dashed line).
