# Supplementary material for: Detectability in Audio-Visual Surveys of Tropical Rainforest Birds: The Influence of Species, Weather and Habitat Characteristics
Source: PLoS One. 2015 Jun 25;10(6):e0128464. doi: 10.1371/journal.pone.0128464 (PMC4482497; doi:10.1371/journal.pone.0128464)
Supplement: S5 Fig — Box-plots on left (Figs A, C) show the median, 25th and 75th quantile of the range of ESW relative differences between treatments, expressed as the proportion of each species’ total ESW. Biplots on the right (Figs B, D) show the distribution among species of shifts in ESW associated with each covariate. N values refer to the number of species compared, an “x” marks those with non-overlapping 95% confidence intervals. For rain (Fig B) these are Mistletoebird (Dicaeum hirundinaceum), and for cluster size (Fig D) Silvereye (Zosterops lateralis). (PDF) [file pone.0128464.s005.pdf]

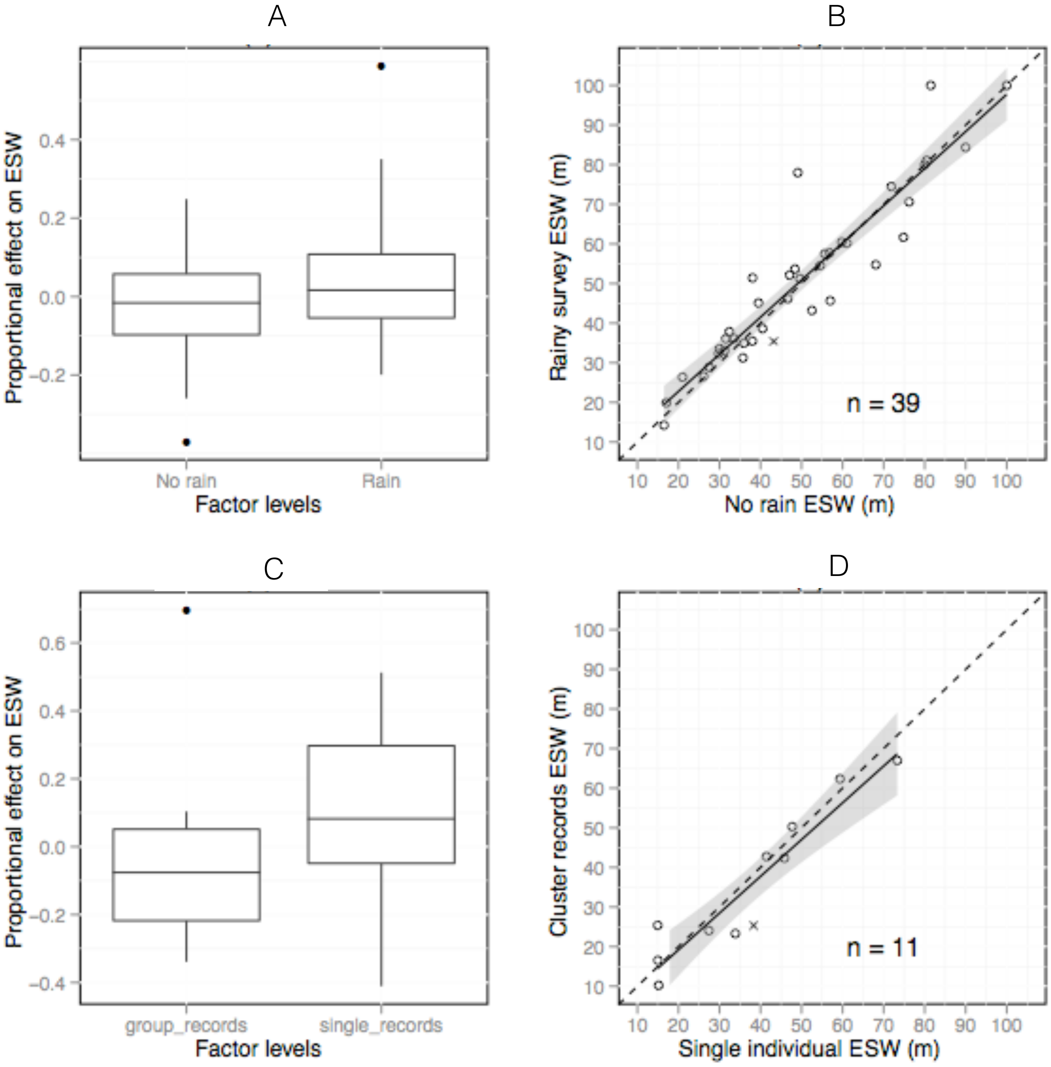

S5 Figs. A comparison of the relative effect of rain and cluster size covariates on Effective Strip Width (ESW). Box-plots on left (Figs. A, C) show the median, 25th and 75th quantile of the range of ESW relative differences between treatments, expressed as the proportion of each species' total ESW. Biplots on the right (Figs. B, D) show the distribution among species of shifts in ESW associated with each covariate. N values refer to the number of species compared, an "x" marks those with non-overlapping 95% confidence intervals. For rain (Fig. B) these are Mistletoebird (*Dicaeum hirundinaceum*), and for cluster size (Fig. D) Silvereye (*Zosterops lateralis*).
