# Supplementary material for: Detectability in Audio-Visual Surveys of Tropical Rainforest Birds: The Influence of Species, Weather and Habitat Characteristics
Source: PLoS One. 2015 Jun 25;10(6):e0128464. doi: 10.1371/journal.pone.0128464 (PMC4482497; doi:10.1371/journal.pone.0128464)
Supplement: S1 Table — These scores were used firstly to guide decisions when to abandon surveys, and secondarily as covariates in the detectability analyses presented here. (PDF) [file pone.0128464.s006.pdf]

S2 Table. Description of the scoring system used to classify survey wind, rain, wetness and noise conditions into relative scores. These scores were used firstly to guide decisions when to abandon surveys, and secondarily as covariates in the detectability analyses presented here.

Factor	Score	Description	Level in analysis
**Wind**	0	Still; leaves not moving	low
	1	Light breeze; leaves rustle	low
	2	Gentle breeze; small twigs moving	high
	3	Moderate breeze; sml branches moving	high
	4	Fresh breeze; sml trees in motion	high
	5	Strong breeze; large branches moving	not surveyed
	6	Near gale; whole trees moving	not surveyed
	7	Gale; falling trees and twigs etc	not surveyed
**Rain**	0	No rain	low
	1	Periodic drizzle	high
	2	Drizzle or periodic light rain	high
	3	Light rain or periodic rain	high
	4	Rain or periodic heavy rain	not surveyed
	5	Continuous heavy rain	not surveyed
**Wet**	0	Dry; soil, litter and leaf surfaces dry to touch	dry
	1	Damp; moisture in soil and/or leaf litter	dry
	2	Wet; water droplets visible on litter and leaf surfaces	wet
	3	Very wet; water droplets visible on litter and falling from leaves, excluding rain	wet
**Noise**	0	No background noise other than bird calls	low
	1	Light or localised background noise (from wind, insects, canopy drip, streams)	high
	2	Loud or more persistent background noise	high
