# Supplementary material for: Detectability in Audio-Visual Surveys of Tropical Rainforest Birds: The Influence of Species, Weather and Habitat Characteristics
Source: PLoS One. 2015 Jun 25;10(6):e0128464. doi: 10.1371/journal.pone.0128464 (PMC4482497; doi:10.1371/journal.pone.0128464)
Supplement: S2 Table — AICc refers to AIC values corrected for small sample size. Density values refer to an estimate per hectare based on the mean counts across all surveys. Model abbreviations are as follows: hn = Half-normal, hr = Hazard rate, uni = Uniform, (see methods, and Thomas et al. (2010) for a detailed description). (PDF) [file pone.0128464.s007.pdf]

S3 Table. Estimates of ESW (Effective Strip Width) by species from Distance sampling and analysis in Australian Wet Tropics (AWT). AICc refers to AIC values corrected for small sample size. Density values refer to an estimate per hectare based on the mean counts across all surveys. Model abbreviations are as follows: hn = Half-normal, hr = Hazard rate, uni = Uniform, (see methods, and Thomas *et al*. (2010) for a detailed description).

#	Species	n	Model (and adjustment)	Effective strip width (ESW) and 95% CI	Density per ha (D) and 95% CI	Probability of detection (P)
1	Australian Brush Turkey (*Alectura lathami*)	35	hr (cos)	15.8 (11.69, 21.44)	0.21 (0.13, 0.34)	0.16
2	Atherton Scrubwren (*Sericornis keri*)	88	hr (poly)	11.3 (7.22, 17.6)	0.92 (0.52, 1.6)	0.11
3	Brown Gerygone (*Gerygone mouki*)	246	uni (cos)	32.86 (30.08, 35.88)	0.88 (0.71, 1.08)	0.33
4	Bridled Honeyeater (*Lichenostomus frenatus*)	154	hr (cos)	44.21 (38.3, 51.03)	0.41 (0.29, 0.57)	0.44
5	Bowers Shrike-Thrush (*Colluricincla boweri*)	114	uni (cos)	43.03 (34.38, 53.85)	0.31 (0.21, 0.45)	0.43
6	Chowchilla (*Orthonyx spaldingii*)	157	uni (cos)	75.05 (66.11, 85.2)	0.25 (0.19, 0.32)	0.75
7	Double-eyed Fig-Parrot (*Cyclopsitta diophthalma*)	82	hr (cos)	55.7 (48.65, 63.74)	0.17 (0.12, 0.25)	0.56
8	Dusky Honeyeater (*Myzomela obscura*)	205	hr (cos)	34.73 (31.98, 37.71)	0.69 (0.55, 0.88)	0.35
9	Emerald Dove (*Chalcophaps indica*)	38	hn (cos)	69.67 (51.1, 94.97)	0.06 (0.04, 0.11)	0.7
10	Eastern Spinebill (*Acanthorhynchus tenuirostris*)	124	hn (cos)	36.67 (27.56, 48.8)	0.4 (0.28, 0.57)	0.37
11	Eastern Whipbird (*Psophodes olivaceus*)	260	hr (cos)	72.89 (65.73, 80.84)	0.42 (0.34, 0.51)	0.73
12	Fairy Gerygone (*Gerygone palpebrosa*)	114	hn (poly)	37.25 (29.61, 46.85)	0.36 (0.26, 0.5)	0.37
13	Figbird (*Sphecotheres vieilloti*)	113	hr (cos)	61.35 (52.86, 71.2)	0.22 (0.15, 0.32)	0.61
14	Fernwren (*Oreoscopus gutturalis*)	154	hn (cos)	30.08 (26.71, 33.89)	0.6 (0.47, 0.77)	0.3
15	Grey Fantail (*Rhipidura albiscapa*)	210	hn (cos)	26.88 (24.34, 29.68)	0.92 (0.74, 1.13)	0.27
16	Graceful Honeyeater (*Meliphaga gracilis*)	538	hn (poly)	41.8 (37.04, 47.18)	1.51 (1.27, 1.8)	0.42

17	Grey-headed Robin (*Heteromyias cinereifrons*)	386	hr (cos)	57.26 (53.7, 61.05)	0.79 (0.68, 0.92)	0.57
18	Golden Whistler (*Pachycephala pectoralis*)	168	hn (cos)	39.04 (30.06, 50.71)	0.5 (0.36, 0.71)	0.39
19	Grey Whistler (*Pachycephala simplex*)	240	hr (cos)	49.38 (45.58, 53.5)	0.57 (0.47, 0.69)	0.49
20	Australian King Parrot (*Alisterus scapularis*)	35	hn (cos)	60.19 (44.44, 81.54)	0.07 (0.04, 0.11)	0.6
21	Little Bronze-Cuckoo (*Chalcites minutillus*)	36	hn (cos)	34.82 (24.4, 49.7)	0.07 (0.04, 0.12)	0.35
22	Large-billed Scrubwren (*Sericornis magnirostra*)	428	hn (poly)	25.64 (23.27, 28.25)	1.96 (1.64, 2.34)	0.26
23	Lewin's Honeyeater (*Meliphaga lewinii*)	132	hr (cos)	56.8 (50.94, 63.35)	0.27 (0.2, 0.37)	0.57
24	Little Shrike-thrush (*Colluricincla megarhyncha*)	594	uni (poly)	48.2 (41.47, 56.03)	1.45 (1.2, 1.74)	0.48
25	Macleay's Honeyeater (*Xanthotis macleayanus*)	279	hr (cos)	54.45 (51.2, 57.9)	0.6 (0.51, 0.7)	0.54
26	Mistletoebird (*Dicaeum hirundinaceum*)	308	hr (poly)	41.28 (38.1, 44.74)	0.88 (0.74, 1.03)	0.41
27	Mountain Thornbill (*Acanthiza katherina*)	186	hr (poly)	18.37 (15.2, 22.21)	1.19 (0.87, 1.63)	0.18
28	Noisy Pitta (*Pitta versicolor*)	89	uni (cos)	56.07 (49.59, 63.39)	0.19 (0.13, 0.26)	0.56
29	Orange-footed Scrubfowl (*Megapodius reinwardt*)	178	hn (cos)	56.15 (45.25, 69.68)	0.37 (0.28, 0.5)	0.56
30	Pied Currawong (*Strepera graculina*)	39	hn (cos)	99.99 (69.16, 144.55)	0.05 (0.03, 0.08)	1
31	Pale-yellow Robin (*Tregellasia capito*)	225	hr (poly)	17.67 (15.51, 20.13)	1.49 (1.21, 1.84)	0.18
32	Rose-crowned Fruit-Dove (*Ptilinopus regina*)	64	hr (cos)	82.9 (66.13, 103.92)	0.09 (0.06, 0.13)	0.83
33	Rainbow Lorikeet (*Trichoglossus haematodus*)	48	hr (cos)	82.82 (61.42, 111.67)	0.07 (0.04, 0.12)	0.83
34	Rufous Fantail (*Rhipidura rufifrons*)	115	hn (poly)	32.9 (26.28, 41.18)	0.41 (0.3, 0.56)	0.33
35	Shining Bronze-Cuckoo (Golden) (*Chalcites lucidus*)	38	uni (cos)	50 (46.2, 54.12)	0.09 (0.06, 0.14)	0.5
36	Sulphur-crested Cockatoo (*Cacatua galerita*)	71	hr (cos)	69.58 (63.3, 76.48)	0.12 (0.08, 0.17)	0.77

37	Spangled Drongo (*Dicrurus bracteatus*)	35	hr (cos)	60.54 (49.51, 74.03)	0.07 (0.04, 0.11)	0.61
38	Silvereye (*Zosterops lateralis*)	382	hr (cos)	32.25 (28.44, 36.57)	1.39 (1.14, 1.7)	0.32
39	Superb Fruit-dove (*Ptilinopus superbus*)	193	hr (cos)	86.67 (75.79, 99.13)	0.26 (0.2, 0.33)	0.87
40	Spotted Catbird (*Ailuroedus melanotis*)	253	hr (cos)	47.52 (44.83, 50.36)	0.62 (0.53, 0.74)	0.48
41	Spectacled Monarch (*Symposiarchus trivirgatus*)	298	hn (cos)	23.85 (21.94, 25.93)	1.47 (1.24, 1.73)	0.24
42	Tooth-billed Bowerbird (*Scenopoeetes dentirostris*)	40	uni (poly)	39.04 (32.17, 47.38)	0.12 (0.08, 0.19)	0.39
43	Topknot Pigeon (*Lopholaimus antarcticus*)	100	uni (poly)	25.81 (20.54, 32.44)	0.45 (0.2, 1.05)	0.26
44	Victoria's Riflebird (*Ptiloris victoriae*)	143	hr (cos)	81.33 (71.93, 91.95)	0.21 (0.17, 0.25)	0.81
45	Varied Triller (*Lalage leucomela*)	162	hr (cos)	59.36 (53.59, 65.74)	0.32 (0.26, 0.39)	0.59
46	White-eared Monarch (*Carterornis leucotis*)	37	hr (cos)	46.28 (37.68, 56.83)	0.06 (0.03, 0.09)	0.46
47	Wompoo Fruit-dove (*Ptilinopus magnificus*)	234	hr (cos)	68.81 (61.59, 76.87)	0.4 (0.32, 0.49)	0.69
48	White-throated Treecreeper (*Cormobates leucophaea*)	166	hr (cos)	52.58 (47.89, 57.72)	0.37 (0.3, 0.46)	0.53
49	Yellow-breasted Boatbill (*Machaerirhynchus flaviventer*)	173	hr (cos)	37.97 (33.35, 43.24)	0.53 (0.42, 0.68)	0.38
50	Yellow Oriole (*Oriolus flavocinctus*)	71	hr (cos)	82.63 (69.3, 98.53)	0.1 (0.07, 0.16)	0.83
51	Yellow-spotted Honeyeater (*Meliphaga notata*)	632	hr (cos)	56.19 (53.42, 59.11)	1.32 (1.16, 1.5)	0.56
52	Yellow-throated Scrubwren (*Sericornis citreogularis*)	184	hr (cos)	16.93 (15.04, 19.07)	1.27 (0.99, 1.64)	0.17
